# Therapeutic efficacy of probiotics for symptoms of attention-deficit hyperactivity disorder in children and adolescents: meta-analysis

**DOI:** 10.1192/bjo.2023.645

**Published:** 2024-01-25

**Authors:** Shun-Chin Liang, Cheuk-Kwan Sun, Chih-Hua Chang, Yu-Shian Cheng, Ruu-Fen Tzang, Hsien-Jane Chiu, Ming Yu Wang, Ying-Chih Cheng, Kuo-Chuan Hung

**Affiliations:** Department of Management Center, Jianan Psychiatric Center, Ministry Of Health and Welfare, Taiwan; Department of Center for General Education, University of Kun Shan, Taiwan; and Department of Optometry, University of Chung Hwa of Medical Technology, Taiwan; Department of Emergency Medicine, E-Da Dachang Hospital, I-Shou University, Taiwan; and School of Medicine for International Students, College of Medicine, I-Shou University, Taiwan; Department of Psychiatry, Tsyr-Huey Mental Hospital, Kaohsiung Jen-Ai's Home, Taiwan; Department of Psychiatry, Mackay Memorial Hospital, Taiwan; Taoyuan Psychiatric Center, Ministry of Health and Welfare, Taiwan; and Institute of Hospital and Health Care Administration, National Yang-Ming University, Taiwan; Department of Psychiatry, China Medical University Hsinchu Hospital, China Medical University, Taiwan; and Department of Health Services Administration, China Medical University, Taiwan; Department of Psychiatry, China Medical University Hsinchu Hospital, China Medical University, Taiwan; Institute of Epidemiology and Preventive Medicine, College of Public Health, National Taiwan University, Taiwan; and Research Center of Big Data and Meta-analysis, Wan Fang Hospital, Taipei Medical University, Taiwan; Department of Anesthesiology, Chi Mei Medical Center, Taiwan

**Keywords:** Probiotics, attention-deficit hyperactivity disorder, meta-analysis, attention, neurodevelopment

## Abstract

**Background:**

The efficacy of probiotics as a therapeutic alternative for attention-deficit hyperactivity disorder (ADHD) remain unclear.

**Aims:**

To investigate the effectiveness of probiotics for symptoms of ADHD and identify possible factors affecting their efficacy.

**Method:**

Randomised placebo-controlled trials were identified through searching major databases from inception to April 2023, using the main keywords ‘probiotics’ and ‘ADHD’ without limitation on languages or geographic locations. The outcome of interest included improvement in total symptoms of ADHD, symptoms of inattention and hyperactivity/impulsivity, and drop-out rate. Continuous and categorical data were expressed as effect sizes based on standardised mean differences (SMDs) and odds ratios, respectively, with 95% confidence intervals.

**Results:**

Meta-analysis of seven trials involving 379 participants (mean age 10.37 years, range 4–18 years) showed no significant improvement in total symptoms of ADHD (SMD = 0.25; *P* = 0.12), symptoms of inattention (SMD = 0.14; *P* = 0.3) or hyperactivity/impulsivity (SMD = 0.08; *P* = 0.54) between the probiotic and placebo groups. Despite non-significance on subgroup analyses, there was a large difference in effect size between studies using probiotics as an adjunct to methylphenidate and those using probiotics as supplementation (SMD = 0.84 *v.* 0.07; *P* = 0.16), and a moderate difference in effect size between studies using multiple strains of probiotics and those using single-strain regimens (SMD = 0.45 *v.* 0.03; *P* = 0.19).

**Conclusions:**

Current evidence shows no significant difference in therapeutic efficacy between probiotics and placebos for treatment of ADHD symptoms. However, albeit statistically non-significant, higher therapeutic efficacies associated with multiple-strain probiotics or combining probiotics with methylphenidate may provide direction for further research.

Neurodevelopmental disorders, which include a variety of developmental problems with a common onset early in childhood, often lead to significant impairments in social, personal and academic functioning.^1^ Attention-deficit hyperactivity disorder (ADHD) is among the most common,^2^ with a worldwide prevalence of about 5%.^3^ In addition to the typical behavioural symptoms of inattention and/or hyperactivity/impulsivity,^2^ patients with ADHD have a high rate of comorbidity with other neurodevelopmental disorders.^1^ For instance, up to 65% of children with ADHD may also experience some behavioural symptoms of autism spectrum disorder (ASD).^4^ On the other hand, difficulties in sustaining attention and impulse control, which are typical for patients with ADHD, are also frequently noted in patients with ASD and Tourette disorders.^4,5^ Despite the guideline recommendation of combining pharmacological and behavioural interventions for children diagnosed with ADHD,^6^ management with comorbid disorders in ADHD could be more challenging.^6^ Moreover, notwithstanding the effectiveness of pharmacological interventions for controlling the core symptoms of ADHD,^7^ potential side-effects and stigma toward medication use frequently prevent caregivers of children with ADHD from seeking medical attention.^8^ This may partly explain the increasing popularity of alternative therapies for ADHD,^8^ which were sought by more than 60% of patients in a previous survey.^9^

## Probiotics for ADHD

Probiotics, which may contain different strains of microorganisms considered to offer health benefits,^10^ have been prescribed for a variety of neurodevelopmental disorders.^11^ The mechanism underlying the therapeutic potential of probiotics lies in the gut–brain axis, which serves as a route for bidirectional communication through neuroendocrinological pathways,^12^ and involves modulation of important neurotransmitters^13,14^ and suppression of inflammation.^15,16^ Nevertheless, the results of previous investigations into the therapeutic benefits of probiotics in the treatment of ADHD symptoms remain inconsistent.^17–23^ Among the four randomised controlled trials (RCTs) that reported an improvement in ADHD symptoms after probiotic treatment,^17,18,21,23^ only two showed a significantly better therapeutic effect in the probiotic group than the placebo group.^18,23^ Variations in other factors, including differences in the number of probiotic strains used and whether probiotics were given as supplementation or an adjunct to psychostimulants, may also influence their therapeutic potency.^17–23^ However, there was no pooled evidence addressing either the therapeutic efficacy of probiotics for symptoms of ADHD or the potential factors that may influence their therapeutic efficacy.

## The current study

Therefore, this meta-analysis was aimed at studying the effectiveness of probiotics for symptoms of ADHD, and identifying possible factors that may affect their therapeutic efficacy.

## Method

### Protocol and registration

The current meta-analysis was conducted according to the Preferred Reporting Items for Systematic Reviews and Meta-Analyses (PRISMA) guidelines.^24^ The protocol of this study was registered in the International Prospective Register of Systematic Reviews (PROSPERO; identifier CRD42023409450).

### Search strategy and selection criteria

Major databases, including PubMed, EMBASE, Cochrane CENTRAL, ScienceDirect, and Clinicaltrials.gov, were searched for RCTs investigating the efficacy of probiotics for ADHD treatment from inception to 6 April 2023. Detailed information regarding the keywords used for searching in individual databases is provided in Supplementary Table 1 available at https://doi.org/10.1192/bjo.2023.645. To maximise the scope of our search, we set no restrictions on language and ethnicity, and identified relevant studies from the reference lists of specific reviews or literature. We focused our study on children/adolescents instead of adults, because of variations in presentations of ADHD between the two populations that may require different scoring systems for evaluation.^25,26^ In addition, the suppressing effect of probiotics against central nervous system (CNS) inflammation, which is believed to play a key role in neurodevelopmental disorders,^27,28^ may vary in different stages of development. Our PICO (i.e. population, intervention, comparator and outcomes) criteria for study eligibility were (a) population: RCTs of patients diagnosed with neurodevelopmental disorders, aged <18 years; (b) intervention: probiotics or products including probiotics (i.e. synbiotics) used either as monotherapy or components of combination regimens; (c) comparator: placebo or interventions other than probiotics and (d) outcome: behavioural rating scales for assessing the total symptoms of ADHD and/or symptom subcategories of ADHD, including inattention, hyperactivity and impulsivity. Exclusion criteria were RCTs that did not target children or adolescents, studies using interventions unrelated to probiotics and studies without an outcome assessment for symptoms of ADHD.

### Data extraction and quality assessment

By using the predefined keywords and search strategy (Supplementary Table 1), the titles and abstracts of the identified studies were screened by two independent authors (S.-C.L. and C.-H.C.). The eligibility of the included studies were determined through discussion between the two authors, who were also responsible for independent extraction of information, including publication-specific detail as well as characteristics and outcome data of eligible studies. All disagreements between the two authors were resolved by discussion with a third author (C.-K.S.). Kappa coefficient was used for assessing interrater reliability.^29^ In cases of missing data, we attempted to contact the corresponding authors by email, for original data. Cochrane's ‘risk of bias’ assessment tool^30^ was used to rate the quality of the included studies in accordance with seven main categories: sequence generation, allocation concealment, performance bias, detection bias, attrition bias, reporting bias and other important biases. With regard to the certainty of evidence, the Grading of Recommendations Assessment, Development and Evaluation (GRADE) framework was used to rate the level of evidence for individual outcomes of interest.^31^ Any disagreement on risk of bias and certainty of evidence ratings was settled through discussion with a third author.

### Data synthesis and analysis

Review Manager 5 for Windows (RevMan 5.4; The Nordic Cochrane Center, The Cochrane Collaboration, Copenhagen; 2014; see https://training.cochrane.org/online-learning/core-software/revman) was used for data analysis. The primary outcome of the study was improvement in the total symptoms of ADHD rated by standardised behavioural rating scales. Our secondary outcomes included improvement in the subcategories of ADHD symptoms, including inattention and hyperactivity/impulsivity, as well as treatment acceptability evaluated by comparing the drop-out rates between treatment and comparison groups. Because different behavioural rating scales (i.e. continuous variables) may be used to assess the same outcomes of interest, a random-effects model was chosen for the analysis of our primary outcome and the subgroup analysis of the effect of different therapeutic strategies on treatment outcome. Standardised mean differences (SMDs) with 95% confidence intervals were used to give an overall estimation of the effect size for therapeutic efficacy. As for categorical data, odds ratios with 95% confidence intervals were used. The generic inverse-variance method was chosen for outcomes of continuous variables, and the Mantel–Haenszel method was used for odds ratios. Subgroup analyses focusing on the choice of treatment strategy (i.e. monotherapy versus combination therapy) and the number of probiotic strains (i.e. multiple strains versus single strain) were also performed to identify the potential sources of heterogeneity and factors that may influence the therapeutic outcomes of probiotics. The reliability and robustness of outcomes were evaluated by using leave-one-out sensitivity analysis. For assessment of heterogeneity among the eligible studies, *I*^2^-test was used. A *P*-value <0.05 was used for defining statistical significance for all study outcomes. A funnel plot was inspected to detect potential publication bias, whereas an Egger's regression test was used for the quantitative assessment of publication bias for more than ten sets of data.

## Results

### Study selection and characteristics of included studies

**Fig. 1:**
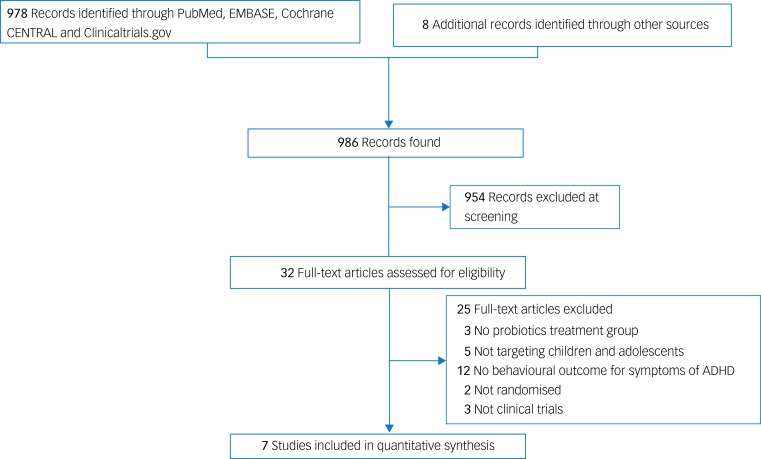
Preferred Reporting Items for Systematic Reviews and Meta-Analyses (PRISMA) diagram of identifying eligible studies. ADHD, attention-deficit hyperactivity disorder.

Figure 1 depicts the process of study selection according to the PRISMA statement.^24^ In short, 978 articles were identified from both published and unpublished databases, and eight additional articles were retrieved from relevant reviews or studies. Following the exclusion of 954 records after screening of titles and abstracts, as well as 25 articles through review of full texts (Supplementary Table 2), seven studies with a total of 397 participants were deemed eligible for study inclusion,^17–23^ and the data from eligible studies were extracted on 10 April 2023. The kappa coefficient was 1, indicating complete agreement among all authors on study eligibility.

Table 1 provides a summary of the characteristics of the included studies. The mean age of the participants was 10.37 years (range: 4–18 years), with a predominance of male participants (female participants: 0–31.6%). Five out of the seven studies recruited patients with a primary diagnosis of ADHD,^18–20,22,23^ one enrolled participants diagnosed with ASD^21^ and the other recruited those with Tourette syndrome.^17^ Three types of rating scales – the Swanson, Nolan, and Pelham (SNAP) scale; the Conners’ Parent Rating Scales – Revised (CPRS-r) and the Disruptive Behavior Disorder (DBD) rating scale – were used for outcome measurement. We compared the ratings from the same type of observers (e.g. parents) for each outcome, to reduce the risk of informant biases between different types of observers. Because only one study used ratings from teachers’ observations,^17^ we were unable to conduct statistical analysis for outcomes from the teacher's perspective. The median duration of treatment was 8 weeks (range: 4–12 weeks). Four out of the seven studies used multiple strains of probiotics, and three used a single strain (Table 1). All studies used the *Lactobacillus* family in their probiotics regimen, with *Lactobacillus plantarum* being most commonly used (i.e. two single-strain probiotic studies and two multiple-strain probiotic studies) (Table 1). Most studies allowed the use of psychotropics except for one,^19^ and two studies recruited participants receiving methylphenidate;^18,23^ information regarding the use of psychotropics was not provided in two studies.^21,22^ The geographic locations of the included trials were diverse, including two in the Middle East,^18,23^ two in Taiwan,^17,21^ two in Europe^19,20^ and one in North America.^22^

**Table 1:** Summary of characteristics of studies in the current meta-analysis

Study (year)	Diagnosis (criteria)	Design	Comparison	*N*	Duration, weeks	Outcome	Medications	Mean age (range), years	Female, %	Country
Ghanaatgar et al (2022)^18^	ADHD (DSM-5)	RCT	Probiotics: 14 strains^a^ plus methylphenidate	25	8	CPRS-r: total	100% methylphenidate	8.8 (6–12)	31.6	Iran
			Placebo plus methylphenidate	25						
Sepehrmanesh et al (2021)^23^	ADHD (DSM-IV-TR)	RCT	Probiotics: 4 strains^b^ plus methylphenidate	20	8	ADHD rating scale	100% methylphenidate	9.1 (8–12)	20.6	Iran
			Placebo plus methylphenidate	20						
Wu et al (2021)^17^	Tourette syndrome (DSM-5)	RCT	Probiotics: *Lactobacillus plantarum*	28	8	SNAP: 1. Total 2. Inattention 3. Hyperactivity/impulsivity	75.4% on different psychotropics (1.8% on methylphenidate)	9.86 (5–18)	15.79	Taiwan
			Placebo	29						
Kumperscak et al (2020)^19^	ADHD (DSM-5)	RCT	Probiotics: *Lactobacillus rhamnosus GG*	18	12	ADHD rating scale	Not allowed psychotropic medications or psychostimulants	11.88 (4–17)	28.13	Europe
			Placebo	14						
Skott et al (2020)^20^	ADHD (DSM-5)	RCT	Probiotics: Synbiotic 2000^c^	65	9	SNAP: 1. Total 2. Inattention 3. Hyperactivity/impulsivity	58.8% on different psychotropics (30.9% on methylphenidate)	12 (median) (5–18)	26.5	Europe
			Placebo	34						
Liu et al (2019)^21^	ASD (DSM-5)	RCT	Probiotics: *Lactobacillus plantarum*	36	4	SNAP: 1. Total 2. Inattention 3. Hyperactivity/impulsivity	Not applicable	10 (7–15)	0	Taiwan
			Placebo	35						
Bazinet (2017)^22^	ADHD and anxiety (DSM-IV)	RCT	Probiotics: *Lactobacillus helveticus* and *Bifidobacterium longum*	25	4	DBD: 1. Total 2. Inattention 3. Hyperactivity/impulsivity	Not applicable	9.75 (6–15)	27.1	Canada
			Placebo	23						

ADHD, attention-deficit hyperactivity disorder; RCT, randomised controlled trial; CPRS-r, Conners’ Parent Rating Scales – Revised; SNAP, Swanson, Nolan, and Pelham scale; ASD, autism spectrum disorder; DBD, Disruptive Behavior Disorder scale.

a *Bacillus subtilis* PXN 21, *Bifidobacterium bifidum* PXN 23, *Bifidobacterium breve* PXN 25, *Bifidobacterium infantis* PXN 27, *Bifidobacterium longum* PXN 30, *Lactobacillus acidophilus* PXN 35, *Lactobacillus delbrueckii* ssp. *bulgaricus* PXN 39, *Lactobacillus casei* PXN 37, *Lactobacillus plantarum* PXN 47, *Lactobacillus rhamnosus* PXN 54, *Lactobacillus helveticus* PXN 45, *Lactobacillus salivarius* PXN 57, *Lactococcus lactis* ssp. *lactis* PXN 63 and *Streptococcus thermophiles* PXN 66.

b *Lactobacillus reuteri*, *Lactobacillus acidophilus*, *Lactobacillus fermentum* and *Bifidobacterium bifidum*.

c Synbiotic 2000 comprises *Lactobacillus plantarum*, *Pediococcus pentosaceus* and *Lactobacillus casei* ssp. *paracasei*.

### Risk-of-bias assessment

**Fig. 2:**
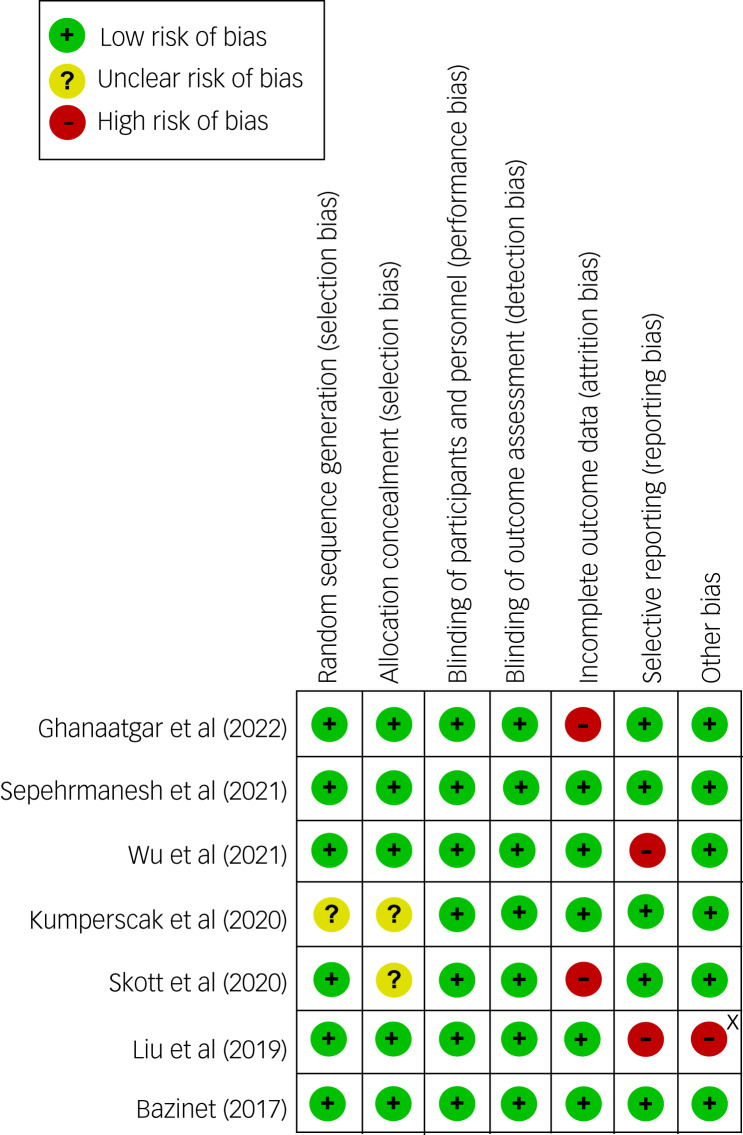
Risk of bias for eligible studies. X, sponsored by a pharmaceutical company.

According to the Cochrane Collaboration's tool for risk-of-bias assessment in randomised trials, most studies had a low risk in detection and performance biases because of their double-blind, placebo-controlled trial design (Fig. 2). However, some studies did not have detailed descriptions about their randomisation process (Fig. 2). Taking into account that two studies had a high risk of reporting bias because they did not primarily focus on ADHD symptoms,^17,21^ the overall quality of evidence could be a concern.

### Primary outcome

**Fig. 3:**
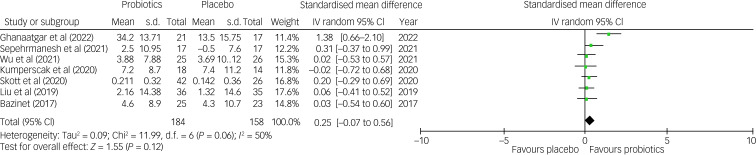
Forest plot of effect size for comparing the difference in the improvement of total symptoms of ADHD between probiotics and placebo groups. ADHD, attention-deficit hyperactivity disorder; IV, inverse variance.

**Fig. 4:**
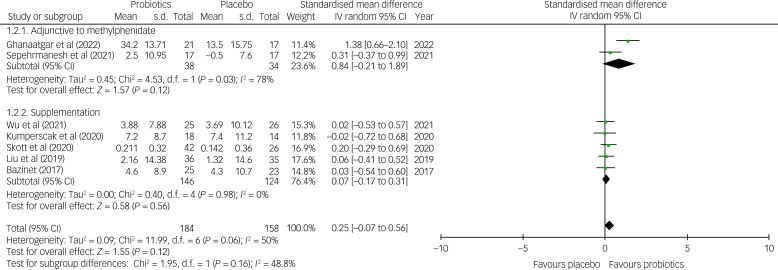
Subgroup analysis showing a forest plot of effect sizes in a subgroup of studies using probiotics as supplementation versus an adjunctive to methylphenidate; IV, inverse variance.

The current meta-analysis showed no significant difference in the improvement of the total symptoms of ADHD between the probiotic and placebo groups (SMD = 0.25, 95% CI −0.07 to 0.56; *P* = 0.12; seven studies with 342 participants) (Fig. 3). Despite no notable asymmetry on funnel pot inspection (Supplementary Fig. 1), there was a trend of heterogeneity on this outcome (*I*^2^ = 50% and *P* = 0.06). Moreover, although robustness assessment with leave-one-out sensitivity test did not reveal any significant change in primary outcome, the heterogeneity of results was substantially reduced (*I*^2^ = 0% and *P* = 0.98) after excluding the trial by Ghanaatgar et al, which was the only study demonstrating a significant improvement in ADHD total symptoms related to treatments with probiotics and methylphenidate compared with methylphenidate treatment alone,^18^ indicating that study was an important source of heterogeneity. Our other subgroup analyses regarding treatment strategies (i.e. supplementation versus adjunct to methylphenidate) and number of probiotic strains (i.e. single versus multiple) did not reveal any significant difference between different subgroups (Fig. 4, Supplementary Figs 2 and 3). Nevertheless, a marked but non-significant difference in effect size was observed between the subgroup of studies using probiotics as an adjunct to methylphenidate and those using probiotics as a supplementation (SMD = 0.84 *v.* 0.07; *P* = 0.16) (Fig. 4). There was also a moderate but non-significant difference in effect size (SMD = 0.45 *v.* 0.03; *P* = 19) regarding the therapeutic effects of probiotics for the treatment of ADHD symptoms between those treated with multiple strains of probiotics and those treated with a single strain (Supplementary Fig. 3).

### Secondary outcomes

The results of secondary outcomes also failed to show significant improvement in the symptoms of inattention (SMD = 0.14, 95% CI −0.12 to 0.38; *P* = 0.3; four studies with 238 participants) (Supplementary Fig. 3) or hyperactivity/impulsivity (SMD = 0.08, 95% CI −0.18 to 0.34; *P* = 0.54; four studies with 238 participants) (Supplementary Fig. 4), compared with the controls. There was no significant heterogeneity, inconsistency on leave-one-out sensitivity analysis or notable asymmetry on funnel plot inspection for both outcomes (Supplementary Figs 5 and 6). There was also no significant difference in the drop-out rate between probiotics and comparison groups (odds ratio 0.99, 95% CI 0.54–1.83; *P* = 0.98; six studies with 361 participants) (Supplementary Figs 7 and 8).

### Certainty of evidence

Supplementary Table 3 provides the details regarding the certainty of evidence for individual outcomes of interest. Given the limited number of eligible trials, problems with heterogeneity and the fact that two studies were not primarily designed for ADHD,^17,21^ the certainty of evidence was rated as very low for the primary outcome. The certainty of evidence was rated as low for secondary outcomes because there were only four studies with data availability, two of which did not focus primarily on the symptoms of ADHD.^17,21^

## Discussion

Despite the proposal of probiotics as a compromising therapeutic regimen for individuals with ADHD,^18,23^ its efficacy remains controversial.^17,18,21,23^ Also, the effects of other factors, such as the use of probiotics as a supplementation or adjunct to psychostimulants, as well as the number of bacterial strains being used, have not been adequately addressed. To the best of our knowledge, our meta-analysis is the first to investigate the efficacy of probiotics for the symptoms of ADHD in children and adolescents. Although our results failed to show superior efficacy of probiotics for the treatment of total or subdomain symptoms of ADHD compared with placebo groups, the limited number of eligible trials cannot rule out the potential therapeutic benefits in this population. In particular, one of the included studies that compared the therapeutic efficacy between participants receiving a combination of methylphenidate with multiple strains of probiotics and those receiving methylphenidate alone, demonstrated a therapeutic benefit of the combined regimen,^18^ suggesting the potential merit of multiple-strain probiotics or the use of probiotics as an adjunct to methylphenidate. Moreover, despite a lack of statistical significance in our findings on subgroup comparisons, larger effect sizes in the subgroups of studies using multiple strains of probiotics or those combining probiotics with methylphenidate may provide a therapeutic insight into the potential adjunctive use of probiotics for ADHD.

The most important hypothesis underlying the relationship between intestinal microbiome and the CNS is the gut–brain axis, through which the gut microbiome and CNS may have bidirectional communication via a variety of neural, endocrine and inflammatory pathways.^12^ There is increasing evidence suggesting the involvement of intestinal microbiota in homeostasis of the entire body, including the CNS.^12,32^ Regarding the possible mechanisms, an animal study demonstrated a possible role of the vagus nerve in the communication between the central gamma-aminobutyric acid (GABA) system and gut microbiome.^33^ Further, the modulation of several important neurotransmitters, including norepinephrine and dopamine,^34^ by certain intestinal microbiota has been reported in both animal and human studies.^13,14^ Also, the anti-inflammatory effects of some probiotics^15,16^ may be beneficial in the treatment of various neurodevelopmental disorders in which systemic and CNS inflammation may play a key role during the developmental period.^27,28^ Previous evidence has shown an association of a perturbation of the microbiome and gut–brain axis with a variety of neurodevelopmental disorders.^11^ Indeed, probiotics have been used as a therapeutic alternative for ADHD in several studies.^17–22^ Nevertheless, despite the finding of a significant improvement in the symptoms of ADHD after treatment with probiotics in previous studies,^17,21^ most failed to demonstrate a significant difference between placebo effects and the therapeutic benefits of probiotics.^17,19–22^ In fact, only one out of the seven studies reported a significant therapeutic efficacy of probiotics compared with placebos.^18^ Another study also showed no difference in therapeutic effects between participants receiving probiotics adjunctive to methylphenidate and those treated with methylphenidate alone,^23^ despite emergence of statistical significance after adjusting for baseline values of age, body mass index and several biochemical variables (e.g. level of high-sensitivity C-reactive protein). Consistent with those findings, our meta-analytic results failed to support a superior therapeutic efficacy of probiotics compared with placebos in the treatment of symptoms of ADHD. On the other hand, taking into account the efficacy of multiple-strain probiotics (up to 14 strains) in one of the included studies,^18^ as well as the significant therapeutic benefit of combining probiotics with methylphenidate compared with methylphenidate alone,^18,23^ it appeared that the efficacy of probiotics for the treatment of ADHD symptoms may be enhanced through a modification of the regimen.

With regard to the association between the composition of gut microbiota and the symptoms of ADHD, previous studies focused primarily on two aspects: (a) comparing differences in microbiome between patients with ADHD and healthy controls, and (b) investigating the diversity of intestinal microflora in ADHD and healthy populations.^35^ However, the results of those studies remain inconsistent, possibly as a result of a variety of methodological issues, including small sample sizes, differences in methods used for microbiome profiling and a lack of control for potential confounding factors such as diet, age and the presence of gastrointestinal diseases.^35^ For instance, the prevalence of *Bifidobacterium* was found to be lower in one report,^35^ but higher in another,^14^ among individuals diagnosed with ADHD than in controls. In contrast, the prevalence of the *Lactobacillus* family, which was the most commonly used strain of probiotics in our included studies, has been shown to be consistently lower in those with ADHD than in their healthy counterparts.^36,37^ Previous animal studies also reported an association of certain members of the *Lactobacillus* family with an increase in neurotransmitter release in the CNS,^38^ as well as improved memory task performance in mice fed with *Lactobacillus* supplementation.^39^ Variations in the choice of probiotic strain and the diversity of microbiomes in the included studies may be two major reasons for the observed heterogeneity in our findings. Although the choice of probiotic strain may not be the major contributor (because all of our included studies used probiotic strains from the *Lactobacillus* family), the correlation between the diversity of microflora and ADHD symptoms remains unclear.^35^ Despite the report of a lower diversity of intestinal microflora in individuals diagnosed with ADHD than in their healthy counterparts in one study,^40^ other studies did not find significant differences between the two groups.^37,41^ In addition, the use of single versus multiple strains may contribute to a difference in efficacy. An *in vitro* study demonstrated that, compared with a single-strain regimen, a combination of multiple strains in probiotic supplementation may enhance its therapeutic benefits through an inhibition of other competing intestinal organisms.^42^ Concordantly, the only study that showed a superior therapeutic effect for the treatment of ADHD symptoms compared with the placebo group in our meta-analysis adopted a multiple-strain probiotic regimen.^18^ In agreement with this finding, a larger effect size reflecting a higher therapeutic efficacy was noted in studies that used multiple strains of probiotics^20,23^ than in those adopting single-strain regimens, despite a lack of statistical significance. Therefore, although current evidence is still not strong enough to support the superior therapeutic effects of the multiple strains of probiotics, the possibility of a more favourable therapeutic outcome associated with the use of multiple-strain probiotic regimens remains, and this requires further elucidation.

Focusing on the impact of therapeutic strategies on the efficacy of probiotics in this clinical setting, a subgroup comparison between the use of probiotics as an adjunct to methylphenidate and its use as supplementation revealed a much larger effect size for use as an adjunct to methylphenidate, despite a lack of statistical significance (SMD = 0.84 *v.* 0.07; *P* = 0.16). Interestingly, the only study that excluded the use of medication for ADHD^19^ also showed the lowest effect size of efficacy for the treatment of ADHD symptoms. The pharmacological effects of methylphenidate for the treatment of ADHD symptoms are primarily derived from a modulation of the dopamine system,^43^ and probiotics may improve neurocognitive functions through their anti-inflammatory effects.^12^ Nevertheless, given the limited number of included studies with non-significant findings, the possibility that probiotics may provide additional benefits through different pathways warrants further investigation.

An analysis of our secondary outcomes not only failed to show better therapeutic effects for probiotics than placebos for the treatment of symptoms of inattention and hyperactivity/impulsivity, but also demonstrated very low effect sizes (i.e. therapeutic efficacies) of probiotics for the two symptom entities (i.e. effect sizes of 0.14 and 0.08, respectively). Nevertheless, none of the studies that provided data for secondary outcome analysis used probiotics as an adjunctive treatment to methylphenidate, and most of them used only one or two strains of microflora in their probiotic regimens.^17,19–22^ Therefore, our findings could not exclude possible therapeutic effects of probiotics for the treatment of symptoms of inattention and hyperactivity/impulsivity when being used as an adjunct to medication or as multiple-strain regimens. Regarding acceptability, the lack of difference in drop-out rate between the treatment and placebo groups indicated satisfactory tolerance of probiotics among individuals with ADHD.

Although we were able to identify seven studies investigating the effects of probiotics for the treatment of ADHD symptoms, a high level of heterogeneity in our primary result underscored the importance of identifying possible confounding factors that may influence the observed therapeutic outcomes. The source of heterogeneity included patient-related factors (i.e. age, gender, dietary patterns, ethnicity),^35^ as well as those related to treatment strategies (i.e. duration of treatment, supplementation or adjunctive use of probiotics, choice of probiotics, number of probiotic strains in the product). Interestingly, although our leave-one-out sensitivity analysis demonstrated a substantial reduction in the level of heterogeneity (*I*^2^ = 0% and *P* = 0.98) after excluding the trial by Ghanaatgar et al,^18^ the exclusion of another study conducted in the same country (Iran) that also used probiotics as an adjunctive to methylphenidate^23^ had no significant impact on heterogeneity. Therefore, the source of heterogeneity from the study by Ghanaatgar et al^18^ may be attributed to its distinctive feature of including up to 14 strains of probiotics in their treatment regimen, taking into account the reported synergistic effects of multiple strains of probiotics through inhibition of other competing intestinal organisms.^42,44^ Such a finding may shed light on a possible direction for future studies focusing on enhancing the therapeutic effectiveness of probiotics for the treatment of ADHD symptoms.

There were several limitations in the present meta-analysis. First, our conclusion, derived from only seven RCTs with a total of 397 participants, was not robust enough to provide solid evidence for clinical practice. In particular, a near-significant heterogeneity in our results may suggest possible sources of heterogeneity. Our subgroup analyses further demonstrated a large difference in effect size between the subgroup of studies using probiotics as an adjunct to methylphenidate and those prescribing probiotics as supplementation, as well as a moderate difference in effect size between the subgroup of studies using multiple-strain probiotics and those adopting single-strain regimens. Further studies are required to elucidate the therapeutic benefits of probiotics, using different therapeutic approaches (e.g. single versus multiple strains). Second, potentially important confounders, such as the use of other nutritional supplements, were not controlled for in most of the included studies. Moreover, because of a lack of relevant information and the limited number of eligible studies, we could not conduct meta-regression to further investigate the effects of other important factors (e.g. age, duration of treatment and country of study) on the therapeutic efficacy of probiotics. Third, a median follow-up duration of merely 8 weeks in our included studies, with none of them having a follow-up period of over 12 weeks, precluded our exploration of the long-term benefits of probiotics in the treatment of ADHD. Fourth, the mean age of 10 years (range: 4–18 years) among the participants limited the extrapolation of our findings to younger children in an active stage of neurological development, whose response to probiotics may be different from that in their older counterparts. In fact, two previous studies reported beneficial effects of an early use of probiotics during the gestational period and early infancy on attentional and cognitive functions.^36,45^ Fifth, despite no obvious asymmetry on the funnel plots, publication bias could not be reliably assessed because of the limited number of eligible trials (fewer than ten), which precluded the conduction of Egger's regression for statistical analysis. Moreover, taking into account previous evidence showing a significant decrease in the effect size of meta-analyses focusing on complementary and alternative medicine after excluding reports in languages other than English,^46^ our results may underestimate the effects of probiotics because of the possibility of language bias. Finally, two out of the seven included studies did not focus primarily on patients with ADHD, which contributed to an increased risk of reporting bias.^17,21^ Overall, given the potential risks of bias and other possible sources of heterogeneity in the present study, further large-scale investigations focusing on patients with ADHD are needed to provide more robust evidence regarding the therapeutic efficacy of probiotics for the treatment of ADHD symptoms.

In conclusion, our study showed no significant difference in therapeutic efficacy between probiotics and placebos for the treatment of ADHD symptoms. However, given the potential sources of heterogeneity and a high risk of attrition bias in some of the included studies, further clinical investigations are warranted to verify our findings. In addition, trends of better therapeutic efficacies observed in certain studies using multiple-strain probiotics or combining probiotics with methylphenidate may provide an avenue for further research to enhance the therapeutic efficacies of probiotics for ADHD.

## Supporting information

Liang et al. supplementary material Liang et al. supplementary material

## Data Availability

The data-sets used and/or analysed during the current study are available from the corresponding author, K.-C.H., on reasonable request.

## References

[1] Morris-Rosendahl DJ, Crocq MA. Neurodevelopmental disorders-the history and future of a diagnostic concept. Dialogues Clin Neurosci 2020; 22: 65–72. 32699506 10.31887/DCNS.2020.22.1/macrocq PMC7365295

[2] Zhou R, Xia Q, Shen H, Yang X, Zhang Y, Xu J. Diagnosis of children's attention deficit hyperactivity disorder (ADHD) and its association with cytomegalovirus infection with ADHD: a historical review. Int J Clin Exp Med 2015; 8: 13969–75. 26550354 PMC4613039

[3] Polanczyk G, de Lima MS, Horta BL, Biederman J, Rohde LA. The worldwide prevalence of ADHD: a systematic review and metaregression analysis. Am J Psychiatry 2007; 164: 942–8. 17541055 10.1176/ajp.2007.164.6.942

[4] Sokolova E, Oerlemans AM, Rommelse NN, Groot P, Hartman CA, Glennon JC, et al. A causal and mediation analysis of the comorbidity between attention deficit hyperactivity disorder (ADHD) and autism spectrum disorder (ASD). J Autism Dev Disord 2017; 47: 1595–604. 28255761 10.1007/s10803-017-3083-7 PMC5432632

[5] Oluwabusi OO, Parke S, Ambrosini PJ. Tourette syndrome associated with attention deficit hyperactivity disorder: the impact of tics and psychopharmacological treatment options. World J Clin Pediatr 2016; 5: 128–35. 26862512 10.5409/wjcp.v5.i1.128 PMC4737687

[6] Wolraich ML, Hagan JF Jr, Allan C, Chan E, Davison D, Earls M, et al. Clinical practice guideline for the diagnosis, evaluation, and treatment of attention-deficit/hyperactivity disorder in children and adolescents. Pediatrics 2019; 144(4): e20192528. 31570648 10.1542/peds.2019-2528 PMC7067282

[7] Rappley MD. Attention deficit–hyperactivity disorder. N Engl J Med 2005; 352: 165–73. 15647579 10.1056/NEJMcp032387

[8] Searight HR, Robertson K, Smith T, Perkins S, Searight BK. Complementary and alternative therapies for pediatric attention deficit hyperactivity disorder: a descriptive review. ISRN Psychiatry 2012; 2012: 804127. 23762770 10.5402/2012/804127 PMC3671686

[9] Stubberfield T, Parry T. Utilization of alternative therapies in attention-deficit hyperactivity disorder. J Paediatr Child Health 1999; 35: 450–3. 10571757 10.1046/j.1440-1754.1999.355401.x

[10] Hill C, Guarner F, Reid G, Gibson GR, Merenstein DJ, Pot B, et al. The international scientific association for probiotics and prebiotics consensus statement on the scope and appropriate use of the term probiotic. Nat Rev Gastroenterol Hepatol 2014; 11: 506–14. 24912386 10.1038/nrgastro.2014.66

[11] Ligezka AN, Sonmez AI, Corral-Frias MP, Golebiowski R, Lynch B, Croarkin PE, et al. A systematic review of microbiome changes and impact of probiotic supplementation in children and adolescents with neuropsychiatric disorders. Prog Neuropsychopharmacol Biol Psychiatry 2021; 108: 110187. 33271210 10.1016/j.pnpbp.2020.110187 PMC8138744

[12] Cryan JF, Dinan TG. Mind-altering microorganisms: the impact of the gut microbiota on brain and behaviour. Nat Rev Neurosci 2012; 13: 701–12. 22968153 10.1038/nrn3346

[13] Asano Y, Hiramoto T, Nishino R, Aiba Y, Kimura T, Yoshihara K, et al. Critical role of gut microbiota in the production of biologically active, free catecholamines in the gut lumen of mice. Am J Physiol Gastrointest Liver Physiol 2012; 303: G1288–95. 23064760 10.1152/ajpgi.00341.2012

[14] Aarts E, Ederveen THA, Naaijen J, Zwiers MP, Boekhorst J, Timmerman HM, et al. Gut microbiome in ADHD and its relation to neural reward anticipation. PLoS One 2017; 12: e0183509. 28863139 10.1371/journal.pone.0183509 PMC5581161

[15] Alipour B, Homayouni-Rad A, Vaghef-Mehrabany E, Sharif SK, Vaghef-Mehrabany L, Asghari-Jafarabadi M, et al. Effects of Lactobacillus casei supplementation on disease activity and inflammatory cytokines in rheumatoid arthritis patients: a randomized double-blind clinical trial. Int J Rheum Dis 2014; 17: 519–27. 24673738 10.1111/1756-185X.12333

[16] Kullisaar T, Songisepp E, Mikelsaar M, Zilmer K, Vihalemm T, Zilmer M. Antioxidative probiotic fermented goats’ milk decreases oxidative stress-mediated atherogenicity in human subjects. Br J Nutr 2003; 90: 449–56. 12908907 10.1079/bjn2003896

[17] Wu CC, Wong LC, Hsu CJ, Yang CW, Tsai YC, Cheng FS, et al. Randomized controlled trial of probiotic PS128 in children with Tourette syndrome. Nutrients 2021; 13(11): 3698. 34835954 10.3390/nu13113698 PMC8619307

[18] Ghanaatgar M, Taherzadeh S, Ariyanfar S, Jahromi S, Martami F, Gharaei J, et al. Probiotic supplement as an adjunctive therapy with ritalin for treatment of attention-deficit hyperactivity disorder symptoms in children: a double-blind placebo-controlled randomized clinical trial. Nutr Food Sci 2022; 53: 19–34.

[19] Kumperscak HG, Gricar A, Ülen I, Micetic-Turk D. A pilot randomized control trial with the probiotic strain Lactobacillus rhamnosus GG (LGG) in ADHD: children and adolescents report better health-related quality of life. Front Psychiatry 2020; 11: 181. 32256407 10.3389/fpsyt.2020.00181 PMC7092625

[20] Skott E, Yang LL, Stiernborg M, Söderström Å, Rȕegg J, Schalling M, et al. Effects of a synbiotic on symptoms, and daily functioning in attention deficit hyperactivity disorder - a double-blind randomized controlled trial. Brain Behav Immun 2020; 89: 9–19. 32497779 10.1016/j.bbi.2020.05.056

[21] Liu YW, Liong MT, Chung YE, Huang HY, Peng WS, Cheng YF, et al. Effects of Lactobacillus plantarum PS128 on children with autism spectrum disorder in Taiwan: a randomized, double-blind, placebo-controlled trial. Nutrients 2019; 11(4): 820. 10.3390/nu11040820 PMC6521002 30979038

[22] Bazinet PA. Effects of probiotics on memory, ADHD, and anxiety in children. *MClinPsychol* Master's thesis, Acadia University, 2017.

[23] Sepehrmanesh Z, Shahzeidi A, Mansournia M, Ghaderi A, Ahmadvand A. Clinical and metabolic reaction to probiotic supplement in children suffering attention-deficit hyperactivity disorder: a randomized, double-blind, placebo-controlled experiment. Int Arch Health Sci 2021; 8: 90.

[24] Moher D, Shamseer L, Clarke M, Ghersi D, Liberati A, Petticrew M, et al. Preferred Reporting Items for Systematic Review and Meta-analysis Protocols (PRISMA-P) 2015 statement. Syst Rev 2015; 4: 1. 25554246 10.1186/2046-4053-4-1 PMC4320440

[25] Green JG, DeYoung G, Wogan ME, Wolf EJ, Lane KL, Adler LA. Evidence for the reliability and preliminary validity of the Adult ADHD Self-Report Scale v1.1 (ASRS v1.1) screener in an adolescent community sample. Int J Methods Psychiatr Res 2019; 28: e1751. 30407687 10.1002/mpr.1751 PMC6877133

[26] Leahy LG. Diagnosis and treatment of ADHD in children vs adults: what nurses should know. Arch Psychiatr Nurs 2018; 32: 890–5. 30454634 10.1016/j.apnu.2018.06.013

[27] Jiang NM, Cowan M, Moonah SN, Petri WA Jr. The impact of systemic inflammation on neurodevelopment. Trends Mol Med 2018; 24: 794–804. 30006148 10.1016/j.molmed.2018.06.008 PMC6110951

[28] Davis RL. Neurodevelopment: inflammation matters. In Advances in Neurotoxicology, Vol. 2 (eds M Aschner, LG Costa): 227–64. Academic Press, 2018.

[29] McHugh ML. Interrater reliability: the kappa statistic. Biochem Med (Zagreb) 2012; 22: 276–82. 23092060 PMC3900052

[30] Higgins JP, Thomas J, Chandler J, Cumpston M, Li T, Page MJ, et al. Cochrane Handbook for Systematic Reviews of Interventions. John Wiley & Sons, 2019. 10.1002/14651858.ED000142 PMC10284251 31643080

[31] Guyatt GH, Oxman AD, Vist GE, Kunz R, Falck-Ytter Y, Alonso-Coello P, et al. GRADE: an emerging consensus on rating quality of evidence and strength of recommendations. BMJ 2008; 336: 924. 18436948 10.1136/bmj.39489.470347.AD PMC2335261

[32] Mayer EA. Gut feelings: the emerging biology of gut-brain communication. Nat Rev Neurosci 2011; 12: 453–66. 21750565 10.1038/nrn3071 PMC3845678

[33] Bravo JA, Forsythe P, Chew MV, Escaravage E, Savignac HM, Dinan TG, et al. Ingestion of Lactobacillus strain regulates emotional behavior and central GABA receptor expression in a mouse via the vagus nerve. Proc Natl Acad Sci U S A 2011; 108: 16050–5. 21876150 10.1073/pnas.1102999108 PMC3179073

[34] Dinan TG, Cryan JF. The impact of gut microbiota on brain and behaviour: implications for psychiatry. Curr Opin Clin Nutr Metab Care 2015; 18: 552–8. 26372511 10.1097/MCO.0000000000000221

[35] Kalenik A, Kardaś K, Rahnama A, Sirojć K, Wolańczyk T. Gut microbiota and probiotic therapy in ADHD: a review of current knowledge. Prog Neuropsychopharmacol Biol Psychiatry 2021; 110: 110277. 33561522 10.1016/j.pnpbp.2021.110277

[36] Pärtty A, Kalliomäki M, Wacklin P, Salminen S, Isolauri E. A possible link between early probiotic intervention and the risk of neuropsychiatric disorders later in childhood: a randomized trial. Pediatr Res 2015; 77: 823–8. 25760553 10.1038/pr.2015.51

[37] Wang LJ, Yang CY, Chou WJ, Lee MJ, Chou MC, Kuo HC, et al. Gut microbiota and dietary patterns in children with attention-deficit/hyperactivity disorder. Eur Child Adolesc Psychiatry 2020; 29: 287–97. 31119393 10.1007/s00787-019-01352-2

[38] Liu WH, Chuang HL, Huang YT, Wu CC, Chou GT, Wang S, et al. Alteration of behavior and monoamine levels attributable to Lactobacillus plantarum PS128 in germ-free mice. Behav Brain Res 2016; 298: 202–9. 26522841 10.1016/j.bbr.2015.10.046

[39] Ohland CL, Kish L, Bell H, Thiesen A, Hotte N, Pankiv E, et al. Effects of Lactobacillus helveticus on murine behavior are dependent on diet and genotype and correlate with alterations in the gut microbiome. Psychoneuroendocrinology 2013; 38: 1738–47. 23566632 10.1016/j.psyneuen.2013.02.008

[40] Prehn-Kristensen A, Zimmermann A, Tittmann L, Lieb W, Schreiber S, Baving L, et al. Reduced microbiome alpha diversity in young patients with ADHD. PLoS One 2018; 13: e0200728. 30001426 10.1371/journal.pone.0200728 PMC6042771

[41] Szopinska-Tokov J, Dam S, Naaijen J, Konstanti P, Rommelse N, Belzer C, et al. Investigating the gut microbiota composition of individuals with attention-deficit/hyperactivity disorder and association with symptoms. Microorganisms 2020; 8(3): 406. 10.3390/microorganisms8030406 PMC7143990 32183143

[42] Collado MC, Meriluoto J, Salminen S. In vitro analysis of probiotic strain combinations to inhibit pathogen adhesion to human intestinal mucus. Food Res Int 2007; 40: 629–36.

[43] Faraone SV. The pharmacology of amphetamine and methylphenidate: relevance to the neurobiology of attention-deficit/hyperactivity disorder and other psychiatric comorbidities. Neurosci Biobehav Rev 2018; 87: 255–70. 29428394 10.1016/j.neubiorev.2018.02.001 PMC8063758

[44] Ouwehand A, Invernici M, Furlaneto F, Messora M. Effectiveness of multistrain versus single-strain probiotics: current status and recommendations for the future. J Clin Gastroenterol 2018; 52(suppl 1): S35–40. 29734210 10.1097/MCG.0000000000001052

[45] Slykerman RF, Kang J, Van Zyl N, Barthow C, Wickens K, Stanley T, et al. Effect of early probiotic supplementation on childhood cognition, behaviour and mood a randomised, placebo-controlled trial. Acta Paediatr 2018; 107: 2172–8. 30246890 10.1111/apa.14590

[46] Moher D, Pham B, Lawson ML, Klassen TP. The inclusion of reports of randomised trials published in languages other than English in systematic reviews. Health Technol Assess 2003; 7: 1–90. 10.3310/hta7410 14670218

