# Supplementary material for: Therapeutic efficacy of probiotics for symptoms of attention-deficit hyperactivity disorder in children and adolescents: meta-analysis
Source: BJPsych Open. 2024 Jan 25;10(1):e36. doi: 10.1192/bjo.2023.645 (PMC10897698; doi:10.1192/bjo.2023.645)

**eTable 1. Applied keywords and the search results from each database**

| Database | Keywords | Filter | Date (yyyy/mm/dd) | Result |
|---|---|---|---|---|
| PubMed | (adhd OR hkd OR add OR Attention deficit hyperactivity disorder OR attention deficit OR hyperactivity OR inattentive OR impulsivity OR neurodevelopmental disorder) and (probiotics OR gut microbiota OR gut-brain axis OR bifidobacteria OR lactobacillus) | RCT | 2023/04/06 | 45 |
| Embase | (adhd OR hkd OR add OR Attention deficit hyperactivity disorder OR attention deficit OR hyperactivity OR inattentive OR impulsivity OR neurodevelopmental disorder) and (probiotics OR gut microbiota OR gut-brain axis OR bifidobacteria OR lactobacillus) | RCT | 2023/04/06 | 440 |
| Cochrane CENTRAL | (adhd OR hkd OR add OR Attention deficit hyperactivity disorder OR attention deficit OR hyperactivity OR inattentive OR impulsivity OR neurodevelopmental disorder) and (probiotics OR gut microbiota OR gut-brain axis OR bifidobacteria OR lactobacillus) | Trials | 2023/04/06 | 181 |
| ScienceDirect | (Attention deficit hyperactivity disorder OR inattentive OR neurodevelopmental | Research | 2023/04/06 | 306 |

| | | | | |
|---|---|---|---|---|
| | disorder) and (probiotics) | articles | | |
| ClinicalTrials.gov | (Attention deficit hyperactivity disorder) and (Probiotics) | NA | 2023/04/06 | 6 |

Abbreviations: NA, not applied; RCT, randomized controlled trial

**eTable 2** Reasons for study exclusion

| Reason | Number of excluded studies | References |
|---|---|---|
| No probiotics treatment group | 3 | [1-3] |
| Not targeting children and adolescents | 5 | [4-8] |
| No behavioral outcome for symptoms of ADHD | 12 | [9-20] |
| Not Randomized | 2 | [21, 22] |
| Not clinical trials | 3 | [23-25] |

ADHD Attention deficit hyperactivity disorder

RCT randomized controlled trials

**References**


[1] Wang J, Liu Y, Huang HY, Wu JT, Wang WJ. [Influence of acupuncture on the clinical manifestations and gastrointestinal symptoms of children with autism spectrum disorder]. Zhongguo zhen jiu = Chinese acupuncture & moxibustion. 2022;42(12):1373-6. https://doi.org/10.13703/j.0255-2930.20220111-0004.

[2] Singh I, Behera DP, T KA, Gupta S. Efficacy and safety of tamsulosin vs its combination with mirabegron in the management of lower urinary tract non-neurogenic overactive bladder symptoms (OABS) because of Benign Prostatic Enlargement (BPE)-An open label randomised controlled clinical study. Int J Clin Pract. 2021;75(7):e14184. https://doi.org/10.1111/ijcp.14184.

[3] Smits MM, Fluitman KS, Herrema H, Davids M, Kramer MHH, Groen AK, et al. Liraglutide and sitagliptin have no effect on intestinal

microbiota composition: A 12-week randomized placebo-controlled trial in adults with type 2 diabetes. Diabetes Metab. 2021;47(5):101223. https://doi.org/10.1016/j.diabet.2021.101223.

[4] Zhang X, Yang H, Zhang K, Zhang J, Lu X, Guo H, et al. Effects of exercise or tai chi on Internet addiction in college students and the potential role of gut microbiota: A randomized controlled trial. Journal of affective disorders. 2023;327:404-15. https://doi.org/10.1016/j.jad.2023.02.002.

[5] Schaub AC, Schneider E, Vazquez-Castellanos JF, Schweinfurth N, Kettelhack C, Doll JPK, et al. Clinical, gut microbial and neural effects of a probiotic add-on therapy in depressed patients: a randomized controlled trial. Transl Psychiatry. 2022;12(1):227. https://doi.org/10.1038/s41398-022-01977-z.

[6] Carlos LO, Ramos MRZ, Wagner NRF, Freitas LAC, Felicidade I, Campos ACL. PROBIOTIC SUPPLEMENTATION ATTENUATES BINGE EATING AND FOOD ADDICTION 1 YEAR AFTER ROUX-EN-Y GASTRIC BYPASS: A RANDOMIZED, DOUBLE-BLIND, PLACEBO-CONTROLLED TRIAL. Arquivos brasileiros de cirurgia digestiva : ABCD = Brazilian archives of digestive surgery. 2022;35:e1659. https://doi.org/10.1590/0102-672020210002e1659.

[7] Slykerman RF, Kang J, Van Zyl N, Barthow C, Wickens K, Stanley T, et al. Effect of early probiotic supplementation on childhood cognition, behaviour and mood a randomised, placebo-controlled trial. Acta paediatrica (Oslo, Norway : 1992). 2018;107(12):2172-8. https://doi.org/10.1111/apa.14590.

[8] Cardona D, Roman P, Cañadas F, Sánchez-Labraca N. The Effect of Multiprobiotics on Memory and Attention in Fibromyalgia: A Pilot Randomized Controlled Trial. Int J Environ Res Public Health. 2021;18(7). https://doi.org/10.3390/ijerph18073543.

[9] Yang LL, Stiernborg M, Skott E, Xu J, Wu Y, Landberg R, et al. Effects of a Synbiotic on Plasma Immune Activity Markers and Short-Chain Fatty Acids in Children and Adults with ADHD-A Randomized Controlled Trial. Nutrients. 2023;15(5). https://doi.org/10.3390/nu15051293.

[10] Yamanbaeva G, Schaub AC, Schneider E, Schweinfurth N, Kettelhack C, Doll JPK, et al. Effects of a probiotic add-on treatment on fronto-limbic brain structure, function, and perfusion in depression: Secondary neuroimaging findings of a randomized controlled trial. Journal of affective disorders. 2023;324:529-38. https://doi.org/10.1016/j.jad.2022.12.142.

[11] Sherman HT, Liu K, Kwong K, Chan ST, Li AC, Kong XJ. Carbon monoxide (CO) correlates with symptom severity, autoimmunity, and responses to probiotics treatment in a cohort of children with autism spectrum disorder (ASD): a post-hoc analysis of a randomized controlled trial. BMC Psychiatry. 2022;22(1):536. https://doi.org/10.1186/s12888-022-04151-3.

[12] Li YQ, Sun YH, Liang YP, Zhou F, Yang J, Jin SL. Effect of probiotics combined with applied behavior analysis in the treatment of children with autism spectrum disorder: a prospective randomized controlled trial. Zhongguo Dang Dai Er Ke Za Zhi. 2021;23(11):1103-10. https://doi.org/10.7499/j.issn.1008-8830.2108085.

[13] Zhang L, Xu Y, Li H, Li B, Duan G, Zhu C. The role of probiotics in children with autism spectrum disorders: A study protocol for a randomised controlled trial. PLoS One. 2022;17(2):e0263109. https://doi.org/10.1371/journal.pone.0263109.

[14] Park SK, Kang SB, Kim S, Kim TO, Cha JM, Im JP, et al. Additive effect of probiotics (Mutaflor) on 5-aminosalicylic acid therapy in patients with ulcerative colitis. The Korean journal of internal medicine. 2022;37(5):949-57. https://doi.org/10.3904/kjim.2021.458.

[15] Pärtty A, Kalliomäki M, Wacklin P, Salminen S, Isolauri E. A possible link between early probiotic intervention and the risk of neuropsychiatric disorders later in childhood: a randomized trial. Pediatric research. 2015;77(6):823-8. https://doi.org/10.1038/pr.2015.51.

[16] Chou IC, Kuo HT, Chang JS, Wu SF, Chiu HY, Su BH, et al. Lack of effects of oral probiotics on growth and neurodevelopmental outcomes in preterm very low birth weight infants. J Pediatr. 2010;156(3):393-6. https://doi.org/10.1016/j.jpeds.2009.09.051.

[17] Firmansyah A, Dwipoerwantoro PG, Kadim M, Alatas S, Conus N, Lestarina L, et al. Improved growth of toddlers fed a milk containing synbiotics. Asia Pac J Clin Nutr. 2011;20(1):69-76.

[18] Akar M, Eras Z, Oncel MY, Arayici S, Guzoglu N, Canpolat FE, et al. Impact of oral probiotics on neurodevelopmental outcomes in preterm infants. J Matern Fetal Neonatal Med. 2017;30(4):411-5. https://doi.org/10.1080/14767058.2016.1174683.

[19] Billeci L, Callara AL, Guiducci L, Prosperi M, Morales MA, Calderoni S, et al. A randomized controlled trial into the effects of probiotics on electroencephalography in preschoolers with autism. Autism. 2022;27(1):117-32. https://doi.org/10.1177/13623613221082710.

[20] Sanctuary MR, Kain JN, Chen SY, Kalanetra K, Lemay DG, Rose DR, et al. Pilot study of probiotic/colostrum supplementation on gut

function in children with autism and gastrointestinal symptoms. PLoS One. 2019;14(1):e0210064. https://doi.org/10.1371/journal.pone.0210064.

[21] Wang LJ, Yang CY, Kuo HC, Chou WJ, Tsai CS, Lee SY. Effect of Bifidobacterium bifidum on Clinical Characteristics and Gut Microbiota in Attention-Deficit/Hyperactivity Disorder. J Pers Med. 2022;12(2). https://doi.org/10.3390/jpm12020227.

[22] Wallace CJK, Milev RV. The Efficacy, Safety, and Tolerability of Probiotics on Depression: Clinical Results From an Open-Label Pilot Study. Frontiers in Psychiatry. 2021;12.

[23] Wang L, Cai Y, Garssen J, Henricks PAJ, Folkerts G, Braber S. The Bidirectional Gut-Lung Axis in COPD. Am J Respir Crit Care Med. 2023. https://doi.org/10.1164/rccm.202206-1066TR.

[24] Rianda D, Agustina R, Setiawan EA, Manikam NRM. Effect of probiotic supplementation on cognitive function in children and adolescents: a systematic review of randomised trials. Beneficial microbes. 2019;10(8):873-82. https://doi.org/10.3920/bm2019.0068.

[25] Kalenik A, Kardaś K, Rahnama A, Sirojć K, Wolańczyk T. Gut microbiota and probiotic therapy in ADHD: A review of current knowledge. Prog Neuropsychopharmacol Biol Psychiatry. 2021;110:110277. https://doi.org/10.1016/j.pnpbp.2021.110277.

**eTable 3.** Grading of Recommendations Assessments, Development and Evaluation (GRADE) assessment of the strength of evidence for standard weighted meta-analysis

| Outcome | Design | Risk of bias | Indirectness | Inconsistency | Imprecision | Publication bias | Grade Quality |
|---|---|---|---|---|---|---|---|
| Total symptoms | RCT x 7 | No serious | Serious indirectness | Serious inconsistency | No serious imprecision | Serious publication bias | ⊕OOO[2,3,5] |
| Inattention | RCT x 4 | No serious | Serious indirectness | No serious inconsistency | No serious imprecision | Serious publication bias | ⊕⊕OO[2,5] |
| Hyperactivity/impulsivity | RCT x 4 | No serious | Serious indirectness | No serious inconsistency | No serious imprecision | Serious publication bias | ⊕⊕OO[2,5] |
| Numbers of dropout | RCT x 5 | No serious | No serious indirectness | No Serious inconsistency | Serious imprecision | Serious publication bias | ⊕⊕OO[4,5] |

*Risk of bias was estimated using Cochrane risk of bias, studies were classified as having low risk of bias if none of the domains above was rated as high risk of bias and three or less were rated as unclear risk; moderate if one was rated as high risk of bias or none was rated as high risk of bias but four or more were rated as unclear risk, and all other cases were assumed to pertain to high risk of bias.

Down-graded due to: [1] risk of bias, [2] indirectness, [3.] Inconsistency, [4.] Imprecision, [5.] publication bias

**GRADE Working Group grades of evidence:**

-**High certainty**: We are very confident that the true effect lies close to that of the estimate of the effect

-**Moderate certainty**: We are moderately confident in the effect estimate: The true effect is likely to be close to the estimate of the effect, but there is a possibility that it is substantially different.

-**Low certainty**: Our confidence in the effect estimate is limited: The true effect may be substantially different from the estimate of the effect.

-**Very low certainty**: We have very little confidence in the effect estimate: The true effect is likely to be substantially different from the estimate of effect.

**eFigure 1. Funnel plot – ADHD total**

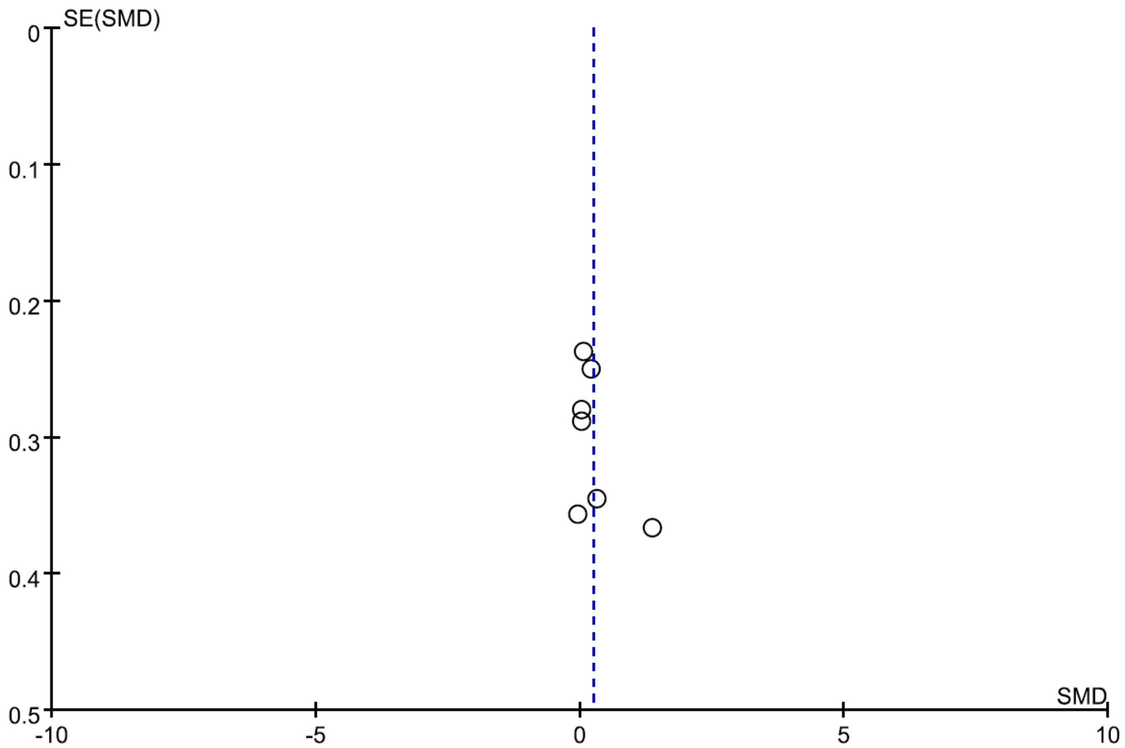

**eFigure 2. Subgroup analysis - forest plot of effect sizes in subgroup of studies using multiple strains of probiotics versus those using a single strain**

| | Probiotics | | | Placebo | | | | Std. Mean Difference | |
|---|---|---|---|---|---|---|---|---|---|
| Study or Subgroup | Mean | SD | Total | Mean | SD | Total | Weight | IV, Random, 95% CI | Year |
| **1.3.1 Mutiple strains** | | | | | | | | | |
| Ghanaatgar M (2022) | 34.2 | 13.71 | 21 | 13.5 | 15.75 | 17 | 11.4% | 1.38 [0.66, 2.10] | 2022 |
| Sepehrmanesh Z (2021) | 2.5 | 10.95 | 17 | -0.5 | 7.6 | 17 | 12.2% | 0.31 [-0.37, 0.99] | 2021 |
| Skott E (2020) | 0.211 | 0.32 | 42 | 0.142 | 0.36 | 26 | 16.9% | 0.20 [-0.29, 0.69] | 2020 |
| Bazinet PA (2017) | 4.6 | 8.9 | 25 | 4.3 | 10.7 | 23 | 14.8% | 0.03 [-0.54, 0.60] | 2017 |
| **Subtotal (95% CI)** | | | **105** | | | **83** | **55.3%** | **0.45 [-0.09, 0.98]** | |
| Heterogeneity: Tau² = 0.20; Chi² = 9.49, df = 3 (P = 0.02); I² = 68% | | | | | | | | | |
| Test for overall effect: Z = 1.62 (P = 0.10) | | | | | | | | | |
| **1.3.2 single strain** | | | | | | | | | |
| Wu CC (2021) | 3.88 | 7.88 | 25 | 3.69 | 10.12 | 26 | 15.3% | 0.02 [-0.53, 0.57] | 2021 |
| Kumperscak HG (2020) | 7.2 | 8.7 | 18 | 7.4 | 11.2 | 14 | 11.8% | -0.02 [-0.72, 0.68] | 2020 |
| Liu YW (2019) | 2.16 | 14.38 | 36 | 1.32 | 14.6 | 35 | 17.6% | 0.06 [-0.41, 0.52] | 2019 |
| **Subtotal (95% CI)** | | | **79** | | | **75** | **44.7%** | **0.03 [-0.29, 0.35]** | |
| Heterogeneity: Tau² = 0.00; Chi² = 0.03, df = 2 (P = 0.98); I² = 0% | | | | | | | | | |
| Test for overall effect: Z = 0.18 (P = 0.86) | | | | | | | | | |
| **Total (95% CI)** | | | **184** | | | **158** | **100.0%** | **0.25 [-0.07, 0.56]** | |
| Heterogeneity: Tau² = 0.09; Chi² = 11.99, df = 6 (P = 0.06); I² = 50% | | | | | | | | | |
| Test for overall effect: Z = 1.55 (P = 0.12) | | | | | | | | | |
| Test for subgroup differences: Chi² = 1.71, df = 1 (P = 0.19), I² = 41.5% | | | | | | | | | |

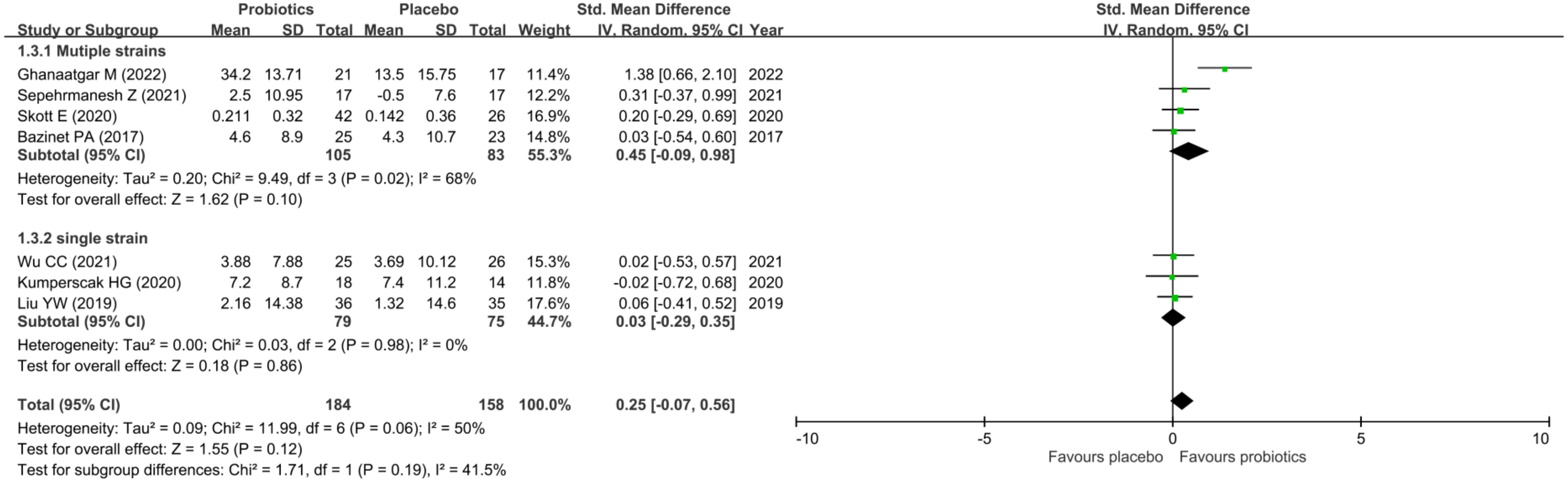

**eFigure 3. Forest plot of effect size for comparing the difference in the improvement of inattention between probiotics and placebo groups**

| | Experimental | | | Control | | | | Std. Mean Difference | Std. Mean Difference |
|---|---|---|---|---|---|---|---|---|---|
| Study or Subgroup | Mean | SD | Total | Mean | SD | Total | Weight | IV, Random, 95% CI | IV, Random, 95% CI |
| Bazinet PA (2017) | -24.1 | 5.5 | 25 | -24 | 5.75 | 23 | 20.5% | -0.02 [-0.58, 0.55] | |
| Liu YW (2019) | 0.79 | 5.87 | 36 | 0.44 | 4.71 | 35 | 30.4% | 0.06 [-0.40, 0.53] | |
| Skott E (2020) | 0.202 | 0.31 | 42 | 0.115 | 0.38 | 26 | 27.3% | 0.25 [-0.24, 0.75] | |
| Wu CC (2021) | 2.04 | 3.27 | 25 | 1.27 | 3.21 | 26 | 21.7% | 0.23 [-0.32, 0.79] | |
| **Total (95% CI)** | | | **128** | | | **110** | **100.0%** | **0.14 [-0.12, 0.39]** | |

Heterogeneity: Tau² = 0.00; Chi² = 0.72, df = 3 (P = 0.87); I² = 0%
Test for overall effect: Z = 1.04 (P = 0.30)

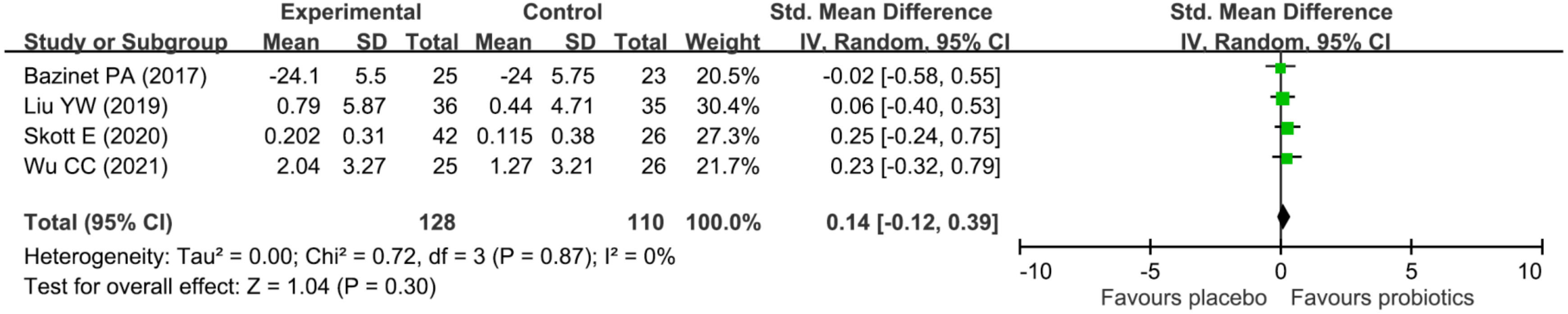

**eFigure 4. Forest plot of effect size for comparing the difference in the improvement of hyperactivity/impulsivity between probiotics and placebo groups**

| | Experimental | | | Control | | | | Std. Mean Difference |
|---|---|---|---|---|---|---|---|---|
| Study or Subgroup | Mean | SD | Total | Mean | SD | Total | Weight | IV, Random, 95% CI |
| Bazinet PA (2017) | -21.4 | 4.5 | 25 | -21.9 | 4.8 | 23 | 20.5% | 0.11 [-0.46, 0.67] |
| Liu YW (2019) | 0.65 | 5.38 | 36 | 0.72 | 6.22 | 35 | 30.4% | -0.01 [-0.48, 0.45] |
| Skott E (2020) | 0.213 | 0.42 | 42 | 0.172 | 0.41 | 26 | 27.4% | 0.10 [-0.39, 0.59] |
| Wu CC (2021) | 1.76 | 3.74 | 25 | 1.12 | 4.09 | 26 | 21.7% | 0.16 [-0.39, 0.71] |
| **Total (95% CI)** | | | **128** | | | **110** | **100.0%** | **0.08 [-0.18, 0.34]** |

Heterogeneity: Tau² = 0.00; Chi² = 0.25, df = 3 (P = 0.97); I² = 0%

Test for overall effect: Z = 0.61 (P = 0.54)

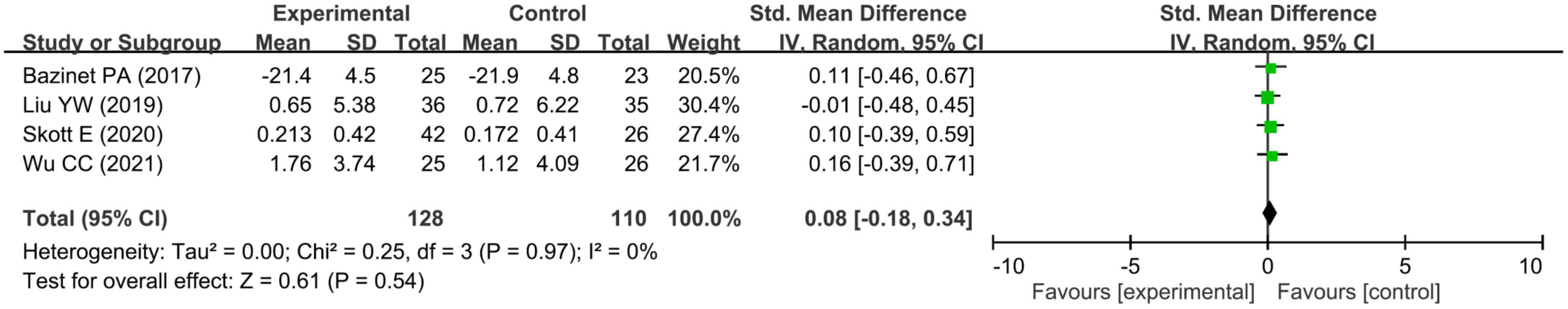

**eFigure 5. Funnel plot – inattention**

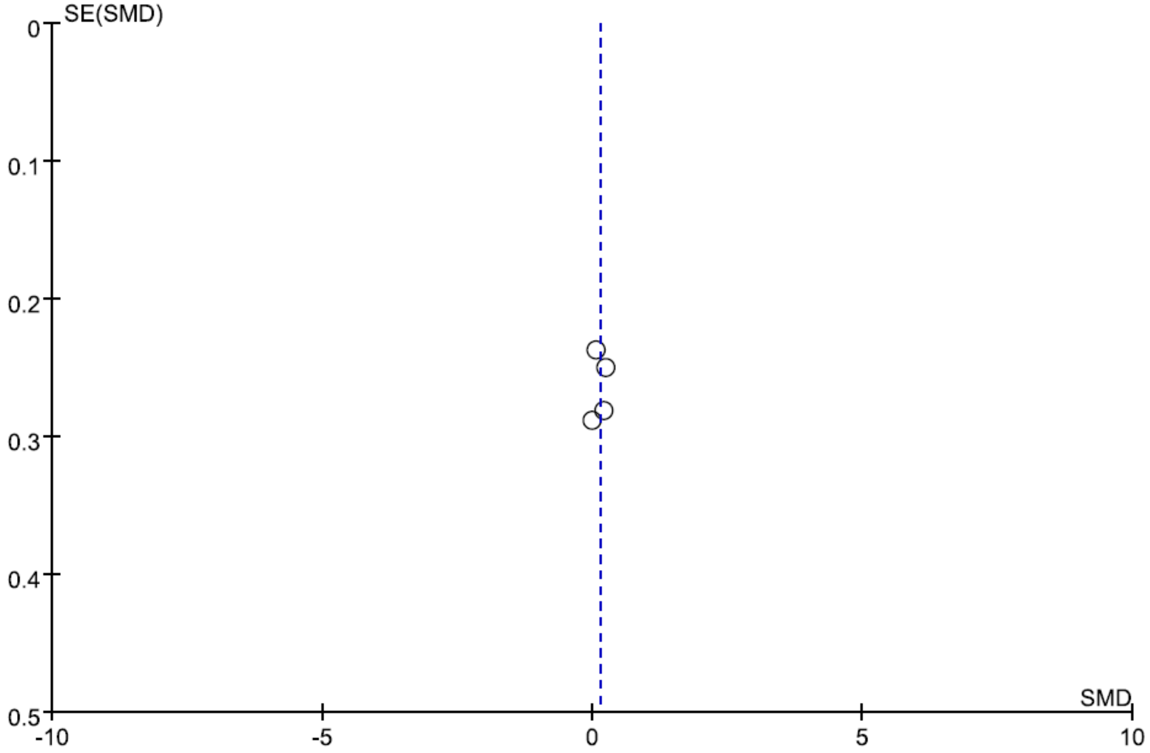

**eFigure 6. Funnel plot – hyperactivity/impulsivity**

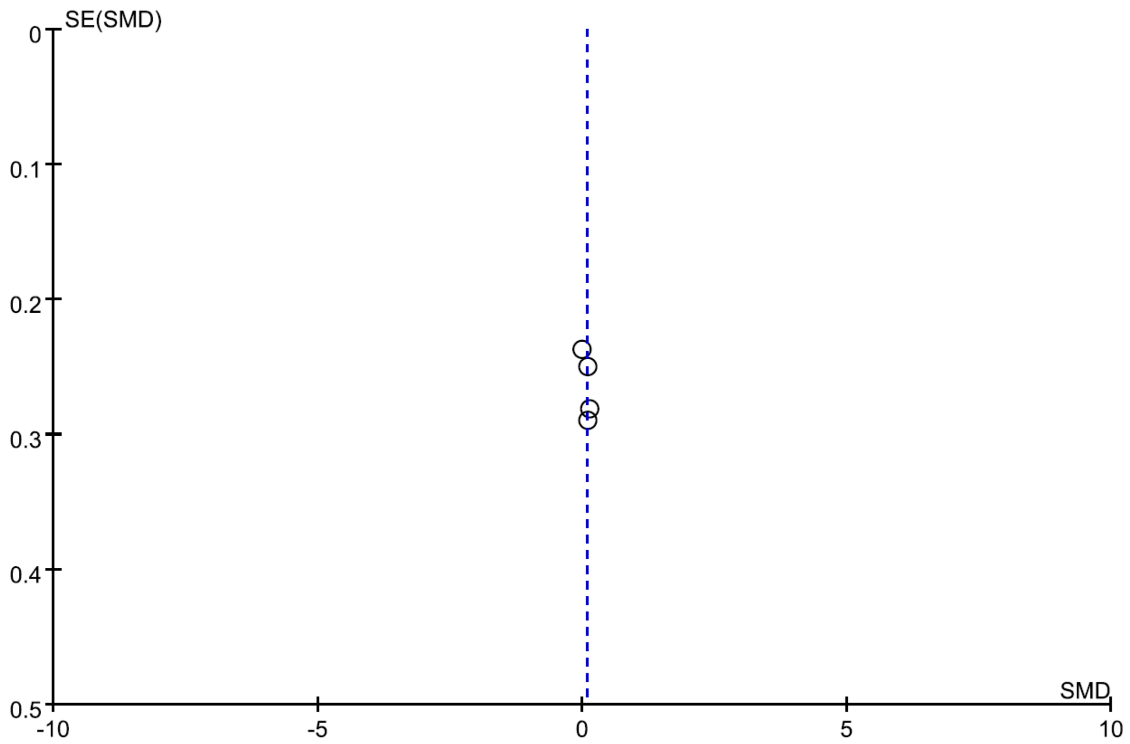

**eFigure 7. Forest plot of odds ratio for comparing the difference in the numbers of dropout between probiotics and placebo groups**

| Study or Subgroup | Probiotics Events | Probiotics Total | Placebo Events | Placebo Total | Weight | Odds Ratio M-H, Fixed, 95% CI | Year |
|---|---|---|---|---|---|---|---|
| Ghanaatgar M (2022) | 4 | 25 | 8 | 25 | 32.6% | 0.40 [0.10, 1.58] | 2022 |
| Sepehrmanesh Z (2021) | 3 | 20 | 3 | 20 | 12.4% | 1.00 [0.18, 5.67] | 2021 |
| Wu CC (2021) | 0 | 28 | 0 | 29 | | Not estimable | 2021 |
| Kumperscak HG (2020) | 0 | 21 | 0 | 14 | | Not estimable | 2020 |
| Skott E (2020) | 23 | 65 | 7 | 34 | 28.8% | 2.11 [0.80, 5.60] | 2020 |
| Liu YW (2019) | 3 | 39 | 6 | 41 | 26.2% | 0.49 [0.11, 2.10] | 2019 |
| **Total (95% CI)** | | **198** | | **163** | **100.0%** | **0.99 [0.54, 1.83]** | |
| Total events | 33 | | 24 | | | | |

Heterogeneity: Chi² = 4.89, df = 3 (P = 0.18); I² = 39%

Test for overall effect: Z = 0.03 (P = 0.98)

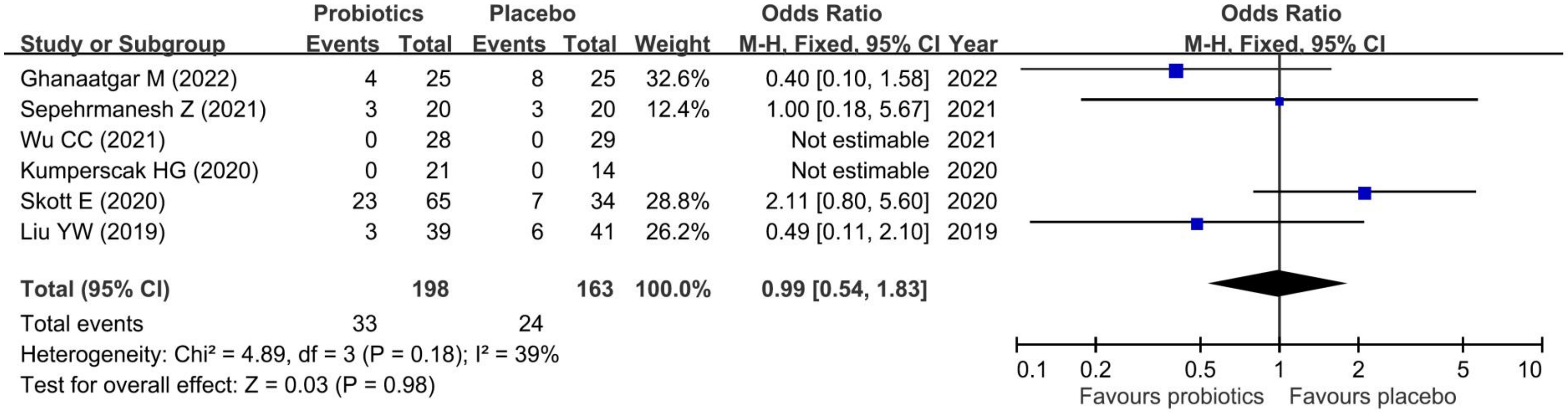

**eFigure 8. Funnel plot – dropout**

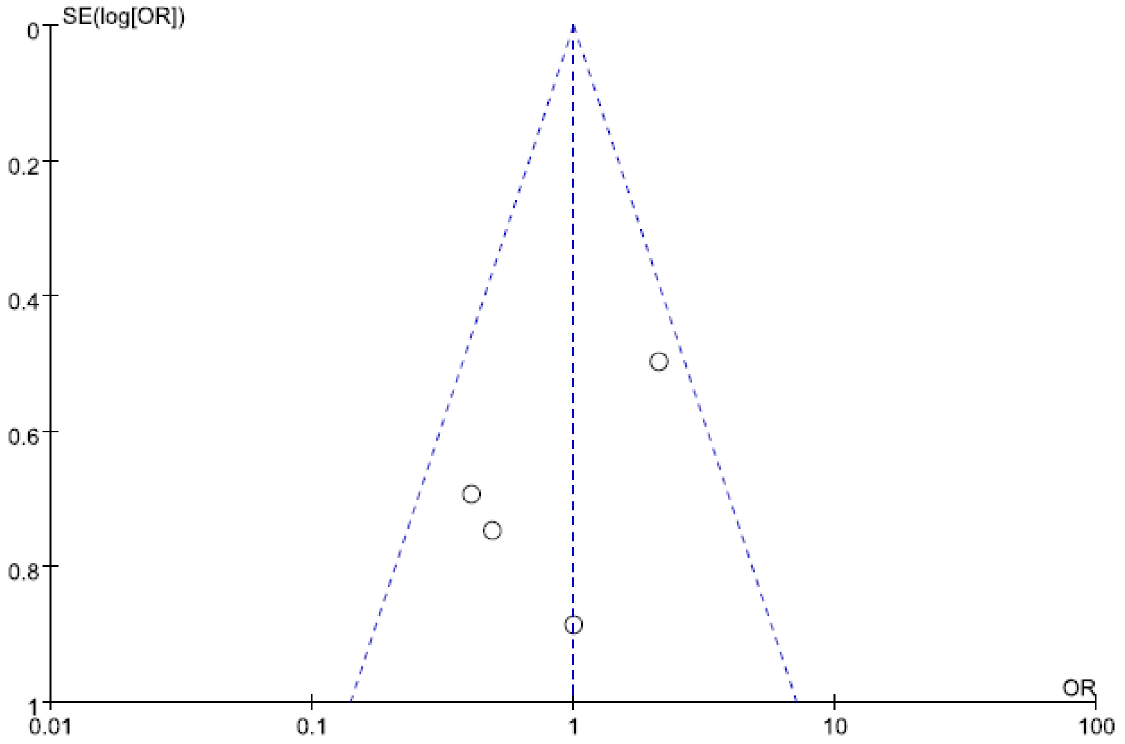

Supplement: Liang et al. supplementary material [file S2056472423006452sup001.pdf]
